# Therapeutic Potential of Intermittent Hypoxia in Atrial Fibrillation

**DOI:** 10.3390/ijms252011085

**Published:** 2024-10-15

**Authors:** Hyewon Park, Bokyeong Park, Kyu-sung Kim, Young Hoon Son, Sung Jin Park, Kichang Lee, Hyelim Park, Junbeom Park

**Affiliations:** 1 Department of Cardiology, College of Medicine, Ewha Womans University School of Medicine, Seoul 07804, Republic of Korea; kiko0615@ewha.ac.kr (H.P.); whitecat0804@naver.com (B.P.); 2 Department of Otorhinolaryngology-Head and Neck Surgery, Inha University School of Medicine, Incheon 22332, Republic of Korea; stedman@inha.ac.kr; 3 Inha Research Institute for Aerospace Medicine, Inha University College of Medicine, Incheon 22332, Republic of Korea; 4 Department of Biomedical Engineering, Emory University School of Medicine, Atlanta, GA 30322, USA; young.hoon.son@emory.edu (Y.H.S.); sung.jin.park@emory.edu (S.J.P.); 5 Cardiovascular Research Center, Massachusetts General Hospital, Boston, MA 02114, USA; kichang.lee@mgh.harvard.edu; 6 Harvard Medical School, Boston, MA 02115, USA

**Keywords:** atrial fibrillation, arrhythmia, Aquaporin 4, intermittent hypoxia, cardiac remodeling

## Abstract

Intermittent hypoxia (IH) has been extensively studied in recent years, demonstrating adverse and beneficial effects on several physiological systems. However, the precise mechanism underlying its cardiac effects on the heart remains unclear. This study aims to explore the effect of treatment on atrial fibrillation under IH conditions, providing data that can potentially be used in the treatment of heart disease. An atrial fibrillation (AF) model was induced by injecting monocrotaline (MCT, 60 mg/kg) into rats. The study included 32 rats divided into four groups: Control, Control + IH, AF, and AF + IH. We evaluated molecular changes associated with AF using ELISA and Western blot and performed electrophysiological experiments to evaluate AF. Arrhythmia-related calcium and fibrosis markers were investigated. Phosphorylation levels of CaMKII, Phospholamban, and RyR2 all increased in the AF group but decreased in the IH-exposed group. Additionally, fibrosis marker expressions such as SMA, MMP2, MMP9, and TGF-β increased in the AF group but were significantly downregulated with IH treatment. Connexin 43 and AQP4 expression were restored in the IH-treated group. These findings suggest that IH may prevent AF by downregulating the expression of calcium-handling proteins and fibrosis-associated proteins in an AF-induced rat model.

## 1. Introduction

Atrial fibrillation (AF) is a prevalent heart arrhythmia often treated with radio-frequency catheter ablation. AF involves structural and electrical remodeling of the heart, which can result from and contribute to the disease. Numerous studies show a higher incidence of AF with increased intermittent hypoxia (IH) severity. Several studies conducted in rodents have shown that IH improves the contractile function of cardiomyocytes and the diastolic function of the heart in ischemic heart disease, suggesting that it may regulate calcium transport [1,2,3,4]. Several proposed mechanisms explain how sleep-disordered breathing, owing to sympathetic nerve hyperactivity, oxidative stress, and inflammation, is associated with AF susceptibility [5,6,7,8].

To understand the impact of IH, it is essential to define important characteristics of the protocol. These potentially include the duration of hypoxia within an episode, the number of hypoxic episodes per day, and the cumulative exposure time. For our study, we used a similarly repeated cycle of hypoxia mixed with normoxic episodes from Russian physicians [9,10,11,12]. The first application of intermittent hypoxia training (IHT) was for athletes, climbers, and pilots. Studies conducted between 1939 and 1943 showed that even a minor increase in height enhances pulmonary ventilation, hemoglobin concentration, and oxygen saturation, highlighting the potential therapeutic effects of hypoxic adaptation. IHT was first acknowledged in sports medicine as a potentially useful strategy to enhance athletic performance. Beyond its benefits in physical performance and preclinical protection, IHT is also considered potentially valuable for enhancing physiological functions in healthy individuals [13]. IHT protects the heart from severe hypoxia by inducing adaptive changes in cardiac structure and function [14,15]. This method holds potential as an emerging therapeutic approach for preventing and treating several diseases—a concept that has garnered growing interest in recent years [3,16,17].

Many studies have reported that intermittent hypoxia treatment can improve major cardiac diseases such as infarct size, ischemic systolic dysfunction, and arrhythmia. In addition, the incidence of coronary artery disease is low in the population living at high altitudes. Therefore, we hypothesized that IH may have a therapeutic effect in rats with atrial fibrillation.

To verify the above hypothesis, we compared the control group treated with normoxia and IH and the atrial fibrillation rat model injected with MCT using electrophysiological analysis and molecular experiments of the heart. We studied the prevention and therapeutic effects of atrial remodeling caused by atrial fibrillation. In addition, these results suggest that it is a potential therapeutic approach for atrial fibrillation.

## 2. Results

### 2.1. Increased Incidence of AF in MCT-Injected Group

**Figure 1:**
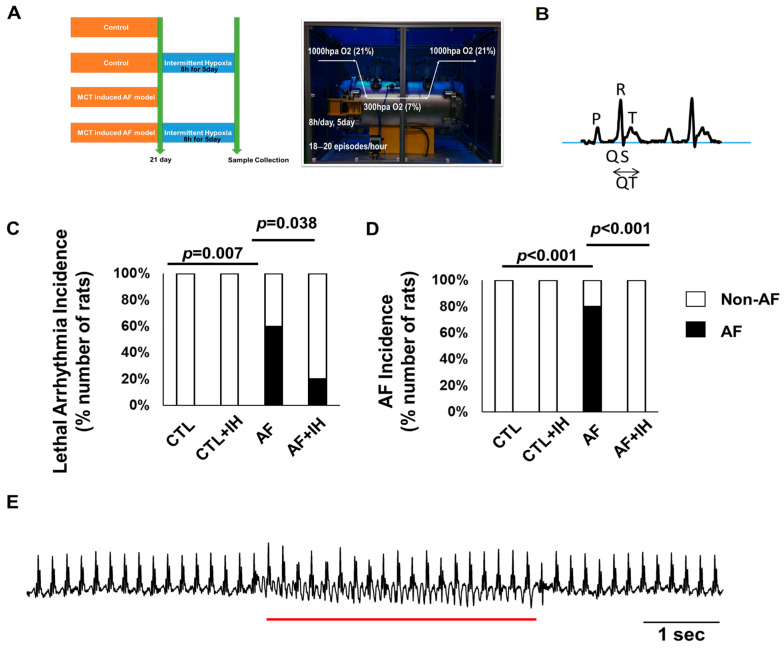
Effect of intermittent hypoxia (IH) exposure on atrial fibrillation (AF) in rats. (**A**) Schematic representation of the design of the animal study and intermittent hypoxia chamber image. (**B**) Representative EKG of a rat at baseline. (**C**) Percentage of lethal arrhythmias observed. (**D**) AF incidence in each group. (**E**) Representative electrocardiogram recording. The underline indicates AF. Control *n* = 8; Control + IH *n* = 8; AF *n* = 8; AF + IH *n* = 8. Statistical analyses: χ2 test for incidence, Student *t*-test, or one-way ANOVA with Bonferroni’s post hoc test for other comparisons.

Figure 1A displays the schematic representation of the animal study and the image of the intermittent hypoxia chamber image. To verify the successful establishment of the rat AF model, electrocardiographic monitoring was conducted (Figure 1B). Figure 1C shows increased arrhythmia occurrences in the AF group (75%) and decreased occurrence in the IH-treated group (25% vs. AF group, *p* = 0.038). AF incidence occurred in four of the five animals with lethal arrhythmia (80%, *p* < 0.001 compared to Control, *p* < 0.001 compared to AF + IH group), and only non-AF arrhythmias occurred in the AF + IH group (Figure 1D). Additionally, changes in P waves and irregular RR intervals, indicative of a persistent AF state, were observed in the rat model (Figure 1D). The IH-treated group showed significantly suppressed heart rate changes in rats. These findings affirm the successful establishment of the rat model for AF.

### 2.2. Effect of IH on Heart Rate Variability

**Figure 2:**
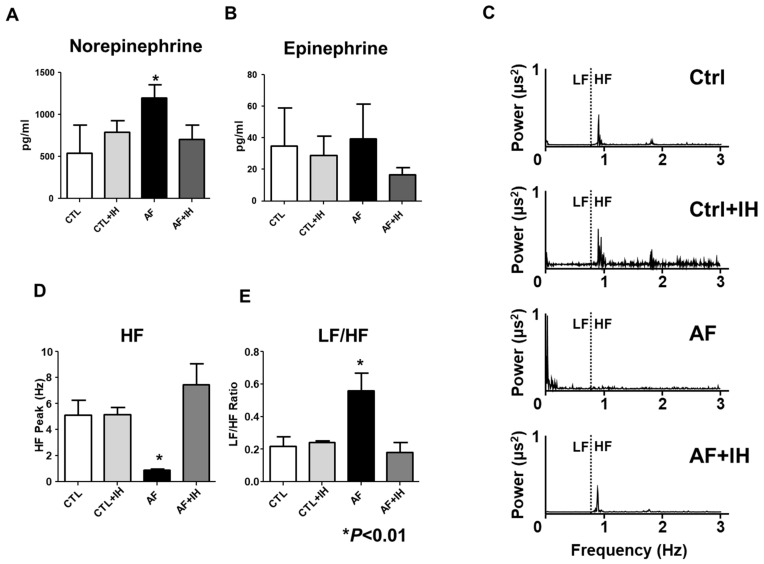
IH effect on HRV. (**A**,**B**) ELISA analysis of serum levels of Norepinephrine and Epinephrine levels across four groups. (**C**) Representative power spectra indicating autonomic controls in cardiac regulation across 5 min segments. (**D**,**E**) Heart rate variability-low frequency (LF) to high frequency (HF) power ratio, denoting cardiac sympathetic activity. Data expressed as mean ± SEM. The data were analyzed using a one-way analysis of variance. * *p* < 0.01 vs. CTL.

The AF group exhibited significantly increased levels of norepinephrine compared to the control group (Figure 2A, 543 ± 277 vs. 1198 ± 260 pg/mL, *p* < 0.001). No significant difference was observed in the epinephrine level between the groups (Figure 2B). IH prevented the AF-induced increases in norepinephrine levels (710 ± 202 pg/mL, *p* < 0.001) (Figure 2B). Figure 2C shows the representative power spectra collected from four groups. Compared to the control, the AF group showed a significantly decreased HF level (5.1 ± 2.6 vs. 0.9 ± 0.2 ms^2^, *p* = 0.007). IH alleviated the AF-induced decrease in HF levels (7.5 ± 3.6 ms^2^, *p* < 0.001). While LF/HF significantly increased in the AF group (0.22 ± 0.13 vs. 0.56 ± 0.25, *p* = 0.026), IH prevented the AF-induced LF/HF increase (0.18 ± 0.13, *p* = 0.017) (Figure 2D,E, Table 1).

### 2.3. IL-6, TNF-α, TGF-β, and MMP2 Measurements

**Figure 3:**
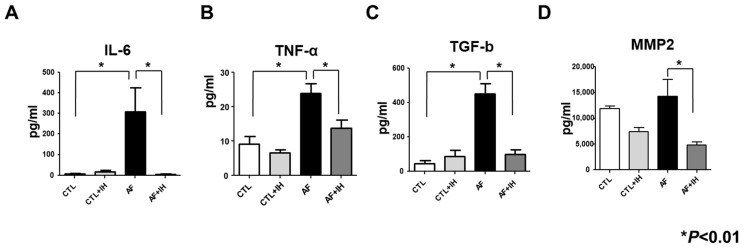
Intermittent hypoxia (IH) suppresses inflammation and fibrosis in atrial fibrillation (AF)-induced rat atrial tissues. (**A**–**D**) Levels of Interleukin-6 (IL-6), Tumor necrosis factor-alpha (TNF-α), Transforming Growth Factor-beta (TGF-B), MMP2. Control *n* = 4; Control + IH *n* = 4; AF *n* = 4; AF + IH *n* = 4. * *p* < 0.01 vs. control group or AF + IH group. Data expressed as mean ± SEM. The data were analyzed using a one-way analysis of variance. * *p* < 0.01 vs. CTL.

ELISA was conducted to determine the IL-6, TNF-α, TGF-β, and MMP2 levels in the serum. The results showed significantly higher IL-6, TNF-α, TGF-β, and MMP2 levels in the serum of the AF group than in the normal control group before IH exposure (*p* < 0.001). After 5 days of IH exposure, serum levels of IL-6, TNF-α, TGF-β, and MMP2 in the AF group significantly decreased than in the AF group (Figure 3A–D, *p* < 0.01).

### 2.4. Effects of IH Administration on CaMKII Activation and Ca^2+^ Handling Disturbances in MCT-Induced AF

**Figure 4:**
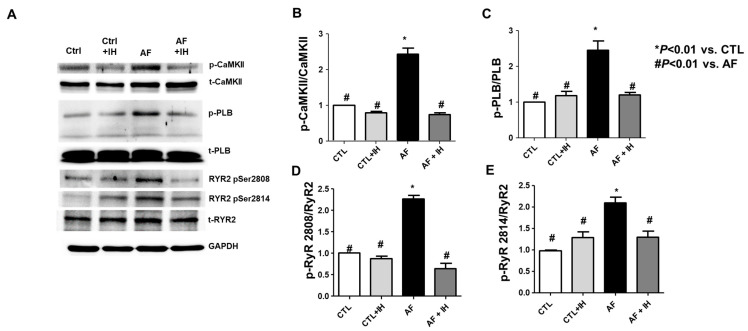
Comparison of Ca^2+^/calmodulin-dependent protein kinase II (CaMKII) and p-CaMKII expression and consequent phosphorylation of downstream targets. Each figure panel shows a (**A**) representative Western blot. (**B**–**E**) Relative expression of phosphorylated and total CaMKII (**B**), Phospholamban (PLB) (**C**), Ryanodine receptor (RyR2) (**D**,**E**). No significant difference was observed in CaMKII, PLB, RyR2 expression, or auto-phosphorylation in the Control and Control + IH groups. Conversely, these factors were increased in the AF group but recovered in the AF + IH group. Data are presented as mean ± SEM of the ratio of normalized phosphorylated protein to total protein extracted from rat hearts (n = 3–4 each). The data were analyzed using a one-way analysis of variance. * *p* < 0.01 vs. CTL. # *p* < 0.01 vs. AF.

CaMKII activation in the heart tissues of AF rats was determined by analyzing phosphorylated-CaMKII (at T287) and total CaMKII protein levels in the heart (Figure 4A).

Figure 4B shows that p-CaMKII significantly increased in the AF heart but decreased to the control group level in the AF + IH group, indicating CaMKII activation under AF conditions (2.4 ± 0.3 vs. 0.7 ± 0.1, *p* < 0.01). Moreover, the p-PLB-to-total-PLB ratio increased in the AF group and was restored in the AF + IH group (Figure 4C, 2.4 ± 0.5 vs. 1.2 ± 0.1, *p* < 0.01).

Changes in CaMKII expression and its activation could influence CaMKII-dependent phosphorylation of RyR2, which encodes a cardiac sarcoplasmic reticulum Ca^2+^ release channel, thereby increasing its opening probability. RyR2 activation was assessed. RyR2 contains several phosphorylation sites, including RyR2-Ser2814 (phosphorylated by CaMKII) and RyR2-Ser2808 (potentially phosphorylated by CaMKII and PKA). Two Phospho-specific antibodies for RyR2-S2814 and RyR2-S2808 were used in Western blots. RyR2 phosphorylation at Ser2814 and Ser2808 showed higher levels in the AF heart than in the control, suggesting increased Ca^2+^ release by RyR2 (Figure 4D, 2.5 ± 0.7 vs. 0.7 ± 0.1, *p* < 0.01, Figure 4E, 2.5 ± 0.4 vs. 0.7 ± 0.1, *p* < 0.01). However, IH treatment significantly attenuated these changes. Additionally, we also examined the total protein levels of RyR2 in these groups, revealing no difference across the groups.

### 2.5. Effect of IH Administration on Cardiac Fibrosis, CX43 Expression, and Cardiac Edema in AF Hearts

**Figure 5:**
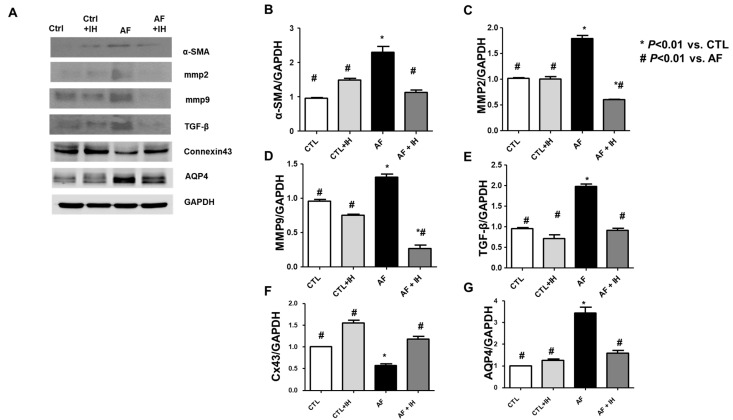
Effect of intermittent hypoxia on fibrosis and water channel protein marker levels in hearts. (**A**) Western blot analysis depicting fibrosis markers α-SMA, MMP2, MMP9, TGF-β, Connexin 43, AQP4, and endogenous control GAPDH. (**B**–**G**) Quantification graphs for each group. Statistical data represent results from four rats in each group. The experimental results are expressed as means ± standard deviations. The data were analyzed using a one-way analysis of variance. * *p* < 0.01 vs. CTL. # *p* < 0.01 vs. AF.

The expression of Cx43, which is crucial for heart electrical conduction and Aquaporin 4 channels, was further analyzed (Figure 5A). First, fibrosis markers affecting cardiac conduction were evaluated. In AF hearts, markers such as MMP2, MMP9, TGF-β, and a-SMA were observed to increase, with relief observed after IH treatment (Figure 5B–E). CX43 expression, vital for heart electrical conduction, underwent further analysis. Significantly downregulated CX43 levels were observed in AF hearts compared to the control group (Figure 4). However, this decrease in CX43 expression was restored with IH treatment (Figure 5F). Finally, the AQP4 expression between the groups was compared, considering its relationship with muscle swelling. Figure 5 illustrates a significant increase in AQP4 expression in AF hearts compared to the control group, whereas its expression decreased in the IH-treated group (Figure 5G).

## 3. Discussion

The main result of this study was that IH administration prevented arrhythmia in AF-induced rat models. Additionally, IH decreased norepinephrine levels, along with the expression of calcium-handling proteins and fibrosis markers in the AF-induced model. These findings suggest that acute IH has beneficial effects on AF. Furthermore, in the MCT-induced AF model, the upregulated expression of calcium-handling proteins and fibrosis markers causes fibrosis and loss of myocardial cells, potentially leading to atrial and ventricular remodeling, heart failure, and malignant arrhythmias. These findings suggest that acute IH exposure could reduce arrhythmia incidence by decreasing the expression of AF-associated proteins in IH-treated AF rat models. However, a limitation of this study is that while various biochemical markers and their content after IH treatment were discussed, the mechanisms involved in the relationship between AF and IH treatment require further investigation.

We assessed the effects of IH on heart rate variability (HRV). HRV represents the fluctuation in the interval between consecutive heartbeats and is produced by heart–brain interactions and dynamic autonomic nervous system activities. Elevated sympathetic activity, along with age-related deterioration in cardiac vagal regulation, disrupts the electrical stability of both the atria and the ventricles, leading to arrhythmogenesis [18].

Low vagally mediated HRV has been suggested as a predictive marker of increased mortality and susceptibility to fatal ventricular arrhythmias [19,20]. On the contrary, interventions (various drugs, exercise training, and vagal nerve stimulation) that enhance vagally mediated HRV are postulated to bring out substantial cardioprotective effects and reduce the risk of SCD [21].

Spectral analysis of HRV data demonstrated comparable findings as IH increased autonomic control (SDNN and total power) with augmentation of the parasympathetic nervous system activity (increased HF and decreased LF/HF) [22]. Thus, we draw a conclusion that IH increases vagally mediated HRV. These findings also validate previous results showing some resemblance in the cardiac actions of IH and beta-blockers. Moreover, in the beta-blocker heart attack trial, the beta-blocker treatment also improved the restoration of parasympathetic tone in patients with acute myocardial infarction as measured by HF power and RMSSD, ultimately leading to improved survival outcomes [23]. We also obtained short-term HRV data on rats subjected to IH. In these studies, similar outcomes were obtained, as IH significantly increased total variability (SDNN). We conclude that IH leads to an increase in vagally mediated HRV, which could play a role in the prevention of arrhythmia.

CaMKII is associated with inflammation, apoptosis, and fibrosis across organ systems [24]. Lin et al. [25] observed a potential role for CaMKII in atrial structural remodeling (Ryanodine receptor-mediated calcium leak drives progressive development of an AF substrate in a transgenic mouse model) as demonstrated in a transgenic mouse model with increased sarcoplasmic reticulum diastolic calcium leak, atrial dilation, and decreased electrical conduction, leading to increased AF susceptibility [26]. Data from Li et al. suggest that CaMKII activity, potentially acting through RYR2 phosphorylation and increased diastolic calcium in the cytoplasm, may contribute to atrial dilation and conduction disturbances [27,28].

The roles of CaMKII, PLB, and RYR2 in an IH-treated AF rat model were examined in this study. In the MCT-induced group, increased calcium release was observed due to increased phosphorylation levels of RYR2 at S2808 and S2814, potentially leading to enhanced spontaneous calcium release in the SR. These could contribute to cardiac arrhythmias. CaMKII activation is often associated with increased phosphorylation of PLB at T17. CaMKII inhibition improves arrhythmias caused by junction ablation under stress conditions. Additionally, no differences were observed in total CaMKII, PLB, and RYR2. All protein phosphorylation levels were restored with IH treatment. Collectively, these findings suggest that CaMKII regulation plays a crucial role in inhibiting AF incidence in IH-treated models.

In this study, decreased Cx43 expression was observed in the MCT-induced group but recovered in the IH-treated group. This indicates the potential role of CaMKII activation in controlling electrical conduction, considering the involvement of Cx43 in cardiac fibrosis and maintaining normal conduction.

AQP4 protein expression was analyzed using Western blotting to understand the mechanism of cardiac edema in the MCT-induced group. AQP4 expression increased in the MCT-induced AF group but significantly decreased in the Control + IH and AF + IH group. These findings support our hypothesis that IH treatment can contribute to the cardioprotective effect and expression of cardiac AQP4 protein could indicate the occurrence of AF. Additionally, given that the cardioprotective mechanism of AQP4 remains incompletely understood, its effects on cardiac electrophysiology, inflammation, and fibrosis markers could represent a novel cardioprotective factor.

In summary, the effects of IH administration on AF-induced rat models were significant, showing decreased expression in Ca^2+^ handling proteins, fibrosis markers, and AQP4. These changes were evaluated and compared after 5 d of IH exposure. Overall, the findings of this study reveal that IH prevents AF by reducing the phosphorylation of calcium-related proteins and the expression of fibrosis markers in AF-induced rat models.

Since our research is for an initial investigation into the effect of IH on AF, there are several limitations that need to be addressed for further study. First, this study confirmed the short-term therapeutic effect with one established IH protocol. Additional studies using long-term treatment under various IH conditions will give insight to confirm the therapeutic effect and optimal treatment conditions in atrial fibrillation. Second, experiments on female rats would allow for more generalization and for identifying the potential gender-specific effects. Third, in addition to molecular and electrophysiological change investigation, adding echocardiography experiments in AF models with heart failure will help us to assess cardiac function. Finally, including an antiarrhythmic drug-treated group can provide the context for the potential clinical relevance of IH treatment and for determining the true therapeutic effect on AF in detail.

## 4. Materials and Methods

### 4.1. Intermittent Hypoxia Condition

A custom-designed, computer-controlled IH system [29] includes a dedicated exposure chamber that generates hypoxic conditions while maintaining consistent temperature, humidity, and airflow. The chamber is equipped to automatically supply oxygen, nitrogen, and carbon dioxide gases as needed. In the Control (CTL) group, 1000 hPa, O_2_—21% (atmospheric oxygen concentrations 21% O_2_; 37 °C) was supplied continuously for 5 days. The AF-induced rats in the IH group were exposed to alternating normoxia (16 h) and hypoxia (8 h). The chambers were set to switch between normoxic (1000 hPa; O_2_—21%) and hypoxic (300 hPa; O_2_—7%) conditions. Each IH cycle lasted 30 s of exposure, occurring 18–20 times per hour. These IH cycles were repeated throughout the 8 h IH period daily for 5 days. The rats were exposed to IH for 8 h per day during the study period.

### 4.2. Model of Right-Sided Heart Disease

All protocols were approved by the Inha University Animal Care and Use Committee in compliance with the Animal Welfare Act and Public Health Service Policy on Humane Care and Use of Laboratory Animals (INHA 231026-898).

Male Sprague Dawley rats weighing between 200 and 275 g were acquired from Orient Bio Inc. (Seoungnam, Republic of Korea). A total of 32 mice were used in the present study. The rats were simultaneously randomized and allocated to control (non-treated) or monocrotaline (MCT) groups (n = 8 per group) without considering any other variables such as weight and size. The MCT animals were administered a single intraperitoneal dose (60 mg/kg) of MCT. On day 21 post-MCT administration, electrocardiogram (ECG) readings were conducted under 2% isoflurane anesthesia to determine AF vulnerability and duration.

### 4.3. Electrocardiogram Telemetry Monitoring

Right before the first MCT administration, male Sprague Dawley rats (8 weeks old) were anesthetized with isoflurane (5% induction, 1.5–3% maintenance) before implanting a rat ECG telemetry device (HD-S11, Data Sciences International, St. Paul, MN, USA) via midline laparotomy. Subsequently, the arterial catheter was inserted into the abdominal aorta and secured with a drop of Vetbond surgical glue and a nitrocellulose patch. The transmitter body was affixed to the abdominal muscle wall using a 5–0 non-absorbable suture. Both ECG leads were tunneled through the abdominal muscle wall caudoventrally and then subcutaneously to achieve a lead II configuration. Rats were administered subcutaneous Buprenex (0.05 mg/kg) before surgery and twice daily for 2 days to manage post-operative pain.

All animals were housed individually in a 12 h dark–light cycle with access to food and water ad libitum. Recordings were conducted in a dedicated animal housing room that remained personnel-free during the recording period. ECGs were captured at 4 kHz, and BP was measured at 500 Hz using Ponemah software 6.51 (Data Sciences International, St. Paul, MN, USA). Continuous 24 h BP/ECG recordings were obtained 3 weeks after implant surgery. The occurrence of spontaneous AF was confirmed by using a telemetry system. The occurrence of AF was defined as a rapid and highly irregular ventricular rate and the absence of a clear P wave on the surface ECG.

### 4.4. Enzyme-Linked Immunosorbent Assay

Blood samples were collected from the abdominal aorta of each rat group on day 21. An enzyme-linked immunosorbent assay (ELISA) was conducted to determine HMGB1, IL-6, TNF-α, norepinephrine, and epinephrine levels in serum. Protein levels in serum were quantified using commercial kits for TGF-b (IBL International, Hamburg, Germany), MMP2 (R&D Systems, Minneapolis, MN, USA), IL-6 (R&D Systems, Minneapolis, MN, USA), TNF-α (R&D Systems, Minneapolis, MN, USA), norepinephrine (MyBioSource, San Diego, CA, USA), and epinephrine (MyBioSource, San Diego, CA, USA), following the manufacturer’s instructions.

### 4.5. Heart Rate Variability (HRV) Analysis

ECG was recorded over a 30 min period using an ECG system (BioAmp FE231, ADInstruments Ltd., Sydney, Australia) and LabChart software (version 8, ADInstruments Ltd.).

The HRV was analyzed in time and frequency domains (spectral analysis was used for frequency domain parameters) with the Kubios (version 3.4.2., Kubios Inc., Kuopio, Finland) software, using the standard frequency-domain for rats: low-frequency band (LF: 0.20–0.75 Hz), and high-frequency band (HF: 0.75–3.00 Hz). Kubios HRV is a heart rate variability analysis software with built-in artifact detection and correction. It is commonly used for HRV analysis, in particular in medical and sports pieces of literature [30]. The LF band of RR interval (LF-RR) and the HF band of RR interval (HF-RR) are reported as absolute values and as normalized units (nu) [31]. Frequency domain analysis was performed by the autoregressive method according to an algorithm developed in our laboratory [32]. The time series were divided into continuous segments of 300 beats, overlapped by half.

### 4.6. Immunoblot Analysis of Ca^2+^ Handling Proteins

Within 5 min after the final IH session, hearts were isolated. Isolated heart tissues were immediately frozen in liquid nitrogen and stored at −80 °C. Total protein samples were extracted from atria using Pierce RIPA buffer (Cat#. 89,900, Thermo Scientific, Waltham, MA, USA) supplemented with 1X protease inhibitor cocktail (GenDEPOT, P3300-001). Protein concentrations were determined using a BSA protein kit, and all protein samples were separated by 10% SDS-PAGE and transferred to polyvinylidene fluoride (PVDF) membranes. The blots were blocked with 5% skim milk for 1 h at room temperature and incubated overnight with primary antibody at 4 °C. The blots were washed, exposed to Horseradish Peroxidase (HRP)-conjugated secondary antibody (1:5000; Santa Cruz Biotechnology, Dallas, TX, USA) for 1h at room temperature, and visualized using an Enhanced Chemiluminescent (ECL) detection kit (ECL Plus, Amersham, Piscataway, NJ, USA). Immunoblots for CaMKII, ryanodine receptor type 2 (RyR2), phospholamban (PLB), and their phosphorylated forms were conducted using the following antibodies: anti-CaMKII and anti-p-CaMKII (1:1000; Santa Cruz Biotechnology); anti-RyR2 (1:1000; Abcam Reagents, Cambridge, UK); anti-p-RyR2 (1:1000; Badrilla, Queen Square, UK); and anti-p-PLB (1:1000; Santa Cruz Biotechnology). Blots were scanned, and band intensities were quantified using Image J 1.52a software (ImageJ, U. S. National Institutes of Health, Bethesda, MD, USA). For analysis, Connexin 43, AQP4, alpha-SMA, MMP9, MMP2 and TGF-beta were normalized against the GAPDH loading control. For other phosphorylated proteins, they were normalized against the respective total protein.

### 4.7. Statistical Analysis

The data collection and analyses were performed by at least 2 independent investigators on coded specimens in a blinded manner. The data were presented as mean ± standard deviation. Statistical comparisons between experimental groups were conducted using unpaired two-tailed Student’s *t*-test or one-way analysis of variance (ANOVA) with Student Newman–Keuls post hoc tests. Statistical analyses were conducted using SPSS version 23.0 (SPSS, Inc., Chicago, IL, USA) and GraphPad Prism 5.01 (GraphPad Software, Inc., La Jolla, CA, USA). A significance level of *p* < 0.05 indicated statistical significance.

## Figures and Tables

**Table 1:** Heart rate variability metrics in time and frequency domains.

Heart Rate Variability	CTL	CTL + IH	AF	AF + IH	*p*-Value between CTL and AF Groups	*p*-Value between AF and AF + IH Groups
SDNN	4.8 ± 2.0	4.7 ± 0.8	1.9 ± 0.5	4.6 ± 3.7	0.034	0.022
lnLF (ms^2^)	1.1 ± 0.73	1.3 ± 0.4	0.5 ± 0.3	1.1 ± 0.8	0.043	0.143
lnHF (ms^2^)	5.1 ± 2.6	5.2 ± 1.3	0.9 ± 0.2	7.5 ± 3.63	0.007	0.003
LF/HF ratio	0.22 ± 0.13	0.24 ± 0.02	0.56 ± 0.25	0.18 ± 0.13	0.026	0.017

SDNN, standard deviation of NN interval; LF, low frequency; HF, high frequency.

## Data Availability

Data are contained within the article.
